# Establishment of local diagnostic reference levels for common adult CT examinations: a multicenter survey in Addis Ababa

**DOI:** 10.1186/s12880-023-00963-1

**Published:** 2023-01-09

**Authors:** Marema Jebessa Kumsa, Teklehaimanot Mezgebe Nguse, Haleluya Biredaw Ambessa, Tesfaye Tefera Gele, Wondemu Geteye Fantaye, Seife Teferi Dellie

**Affiliations:** 1 Department of Medical Radiologic Technology, School of Medicine, College of Health Sciences, Addis Ababa University, Addis Ababa, Ethiopia; 2 Department of Radiology, Tikur Anbessa Specialized Hospital, College of Health Sciences, Addis Ababa University, Addis Ababa, Ethiopia

**Keywords:** Computed Tomography, Diagnostic Reference Levels, Volume CT dose index, Dose-Length Product, Dose optimization, Ethiopia

## Abstract

**Background:**

In medical imaging, a computed tomography (CT) scanner is a major source of ionizing radiation. All medical radiation exposures should be justified and optimized to meet the clinical diagnosis. Thus, to avoid unnecessary radiation doses for patients, diagnostic reference levels (DRLs) have been used. The DRLs are used to identify unusually high radiation doses during CT procedures, which are not appropriate for the clinical diagnosis. It has been successfully implemented in Europe, Canada, Australia, the United States, several industrialized countries, and a few underdeveloped countries. The present study aimed to establish DRLs for the head, chest, and abdominopelvic (AP) CT procedures in Addis Ababa, Ethiopia.

**Methods:**

A pilot study identified the most frequent CT examinations in the city. At the time of the pilot, eighteen CT scan facilities were identified as having functioning CT scanners. Then, on nine CT facilities (50% of functional CT scanners), a prospective analysis of volume CT dose index (CTDI_vol_) and dose length product (DLP) was performed. We collected data for 838 adult patients’ head, chest, and AP CT examinations. SPSS version 25 was used to compute the median values of the DLP and CTDI_vol_ dose indicators. The rounded 75th percentile of CTDI_vol_ and DLP median values were used to define the DRLs. The results are compared to DRL data from the local, regional, and international levels.

**Result:**

The proposed DRLs using CTDI_vol_ (mGy) are 53, 13, and 16 for the head, chest, and AP examinations respectively, while the DLP (mGy.cm) for the respective examinations were 1210, 635, and 822 mGy.cm.

**Conclusion:**

Baseline CT DRLs figures for the most frequently performed in Addis Ababa were provided. The discrepancies in dose between CT facilities and as well as between identical scanners suggests a large potential for dose optimization of examinations. This can be actually achieved through appropriate training of CT technologists and continuous dose audits.

**Supplementary Information:**

The online version contains supplementary material available at 10.1186/s12880-023-00963-1.

## Introduction

Computed Tomography (CT) is a medical imaging modality using specialized x-ray equipment to produce cross-sectional images of the human body, and it is a valuable tool in health care, support in disease screening, diagnosis, treatment, and management of patients [1, 2]. It has been used in diagnostic radiology since the early 1970s and has gained popularity worldwide owing to its substantial and life-saving clinical benefits [3–5]. As CT technology has advanced, the number of medical applications of CT imaging has increased, along with the increased availability and use of CT equipment. However, all these benefits increased population radiation exposure, with a corresponding increased risk of cancer. Therefore, there is increased global attention to appropriately managing ionizing radiation exposures in CT, which requires strict adherence to radiation protection; justification, and optimization; so that the benefit outweighs the risk [6].

The radiation dose received during CT examinations by patients is influenced by a range of factors, including the equipment setting and the protocol used [7]. Failure to adhere to the As Low As Reasonably Achievable principle (ALARA) raises the risk of potential radiation effects.

The international Commission on Radiological Protection (ICRP) introduced the diagnostic reference levels (DRLs) in 1996 and encourages their use in medical imaging utilizing ionizing radiation, including CT examinations [6, 8]. DRLs have been found to be an important tool radiation dose optimization. According to the ICRP [6], DRLs are a form of investigation level used to aid optimization of protection in the medical exposure of patients for diagnostic and interventional procedures. DRLs are used to detect whether the median value of a DRL quantity acquired for a representative group of patients within an established weight range from a specific procedure is unusually high or low for that procedure under routine conditions. A 'DRL value' is a specified numerical value of a DRL quantity that is set at the 75th percentile of the medians of DRL quantity distributions observed at healthcare institutions in a country or region. Establishing DRLs can facilitate dose audits and improve patient radiation protection by promoting a reduction in dose levels without compromising image quality or patient care [9]. The principal aim of optimization is achieving a narrower dose distribution, with lower mean and 75th percentile values [6, 10, 11]. Standardized CT dose indicators used to set up DRLs are the CTDI_vol_ measured in milligray (mGy) and dose-length product (DLP) measured in milligray.centimeter (mGy.cm) [12].

Many countries have established and timely revised DRLs for CT examinations in response to the ICRP's recommendation [13–15]. Despite the fact that many CT scanners have been installed in Addis Ababa over the last decade, no study on the establishment of diagnostic reference values for CT scans in adults has been conducted to our knowledge. The purpose of this study was to develop adult CT diagnostic reference values for head, chest, and AP CT examinations.

## Materials and methods

### Participating institutions

A dose survey was performed on the selected CT scanners in Addis Ababa, Ethiopia's capital city. The Ethiopian Radiation Protection Authority provided a list of CT scanners installed throughout the city. Furthermore, the authority provided us with a letter of support, which allowed us so to approach the facilities with ease. And permission was obtained from the respective facilities to collect data. Then, a pilot study was conducted to identify the actively operating centers and commonly performed CT. We identified eighteen properly functioning CT scanners, eleven of which have enough clients. Unfortunately, two CT scanners did not display the required radiation dose indicators and were thus excluded from the study. Finally, this study was conducted in nine facilities (50% of the city's operational CT scanners) during the study. In keeping with this, the facilities comprise three public hospitals, three private hospitals, and three private diagnostic facilities. Table 1 summarizes the characteristics of each CT scanner. Three CT examinations, with the highest percentage of total examination including head, chest, and AP were chosen for this study.

**Table 1:** CT scanners characteristic

Scanners	Manufacturer	Model	No of slice	Year of installation
1	GE	OPTIMA CT660	64	2018
2	Neusoft	Neuviz16	64	2016
3	Philips	Brilliance CT 64	64	2017
4	Siemens	Somatom Emotions	16	2015
5	Philips	Brilliance CT 64	64	2016
6	Siemens	Somatom Emotions	16	2017
7	Philips	Brilliance CT 64	64	2013
8	GE	OPTIMA CT660	64	2016
*9*	Siemens	Somatom Go	128	2015

Data were collected from 282 patients (158 males and 124 females), 276 patients (136 males and 140 females), and 280 patients (137 males and 143 females) for head, chest, and AP, respectively (see Table 2). For chest and AP, the average weight was 61.78 kg (4.40) and 60.74 kg (3.80), respectively

### Data collection techniques

A survey questionnaire was used to collect data from the selected CT facilities (see Additional file 1: Appendix A). We adopted it from previous works [11, 16]. As per ICRP recommendations [6], thirty or more samples were collected from each institution for each CT examination. From October 8, 2021, to February 29, 2022, we collected data from 838 patients prospectively. The data included patients aged 19 or older and weighing between 40 and 80 kg. For each examination, the manufacturer of the CT scanner, the year of establishment, the patient's sex, age, weight (only for chest and AP), KvP, tube, mAs, scanning range, Volume CT dose index (CTDI_vol_), and dose length product (DLP) have been recorded for examination done without contrast (see Additional file 1: Appendix A). The CTDI_vol_ and DLP values were statistically analyzed since they account for the overall dosimetric information of the examination. Accordingly, the study used three Siemens, three Philips, two GE, and one Neusoft (see Table 1).

### Statistical analysis

IBM SPSS version 25.0 was used to conduct the statistical analysis. Once all the data had been collected, analysis was done to identify and remove outliers. The mean and standard deviation (SD) (mean ± SD) used to express quantitative variables. The descriptive statistics were used to analyze the data. The independent samples t-test with a 95% confidence interval and α-value of 0.05 was used to compare means of private hospitals, diagnostic centers, and public hospitals, as well as dose values within similar CT scanners.

**Fig. 1:**
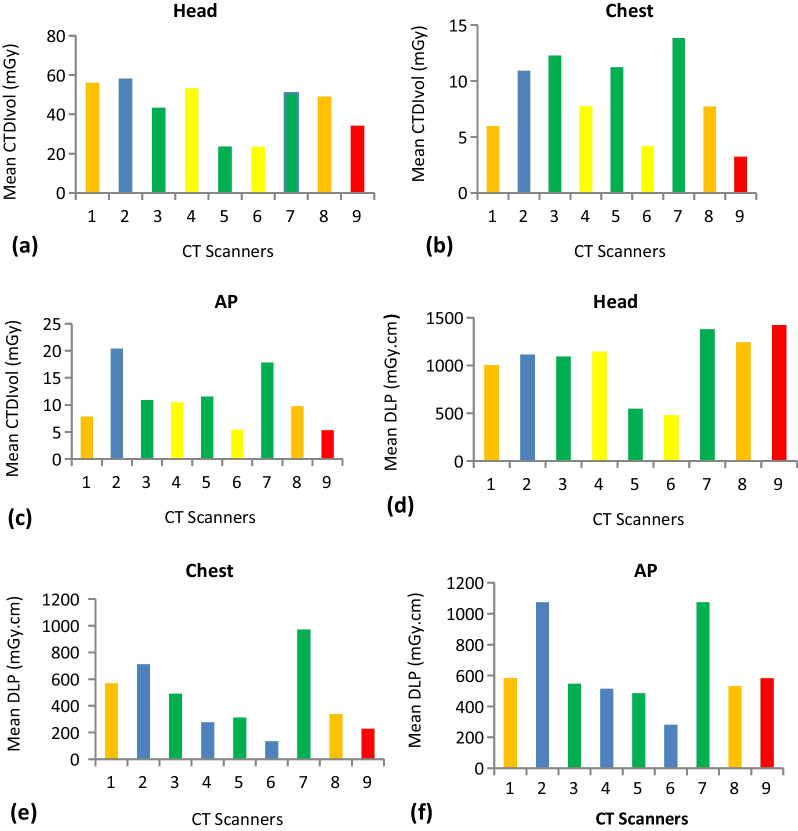
Mean CTDI_vol_ and DLP distribution for examinations surveyed. Graphs depicting the head, chest, and AP mean CTDI_vol_ and DLP. **a** Mean CTDI_vol_ for Head. **b** Mean CTDI_vol_ for chest. **c** Mean CTDI_vol_ for AP. **d** Mean DLP for Head. **e** Mean DLP for chest **f** Mean DLP for AP

**Fig. 2:**
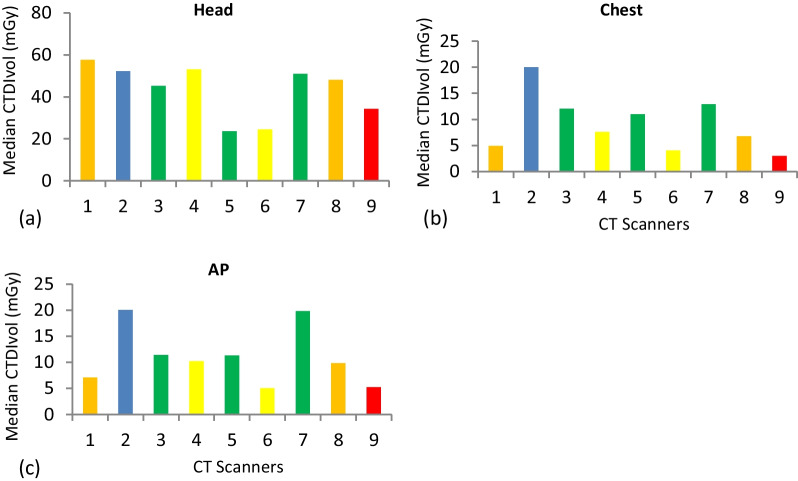
Median CTDI_vol_ distribution for examinations surveyed. Graphs depicting the head, chest, and AP median CTDI_vol_ and DLP. **a** Median CTDI_vol_, for head. **b** Median CTDI_vol_ for chest. **c** Median CTDI_vol_ for AP

**Fig. 3:**
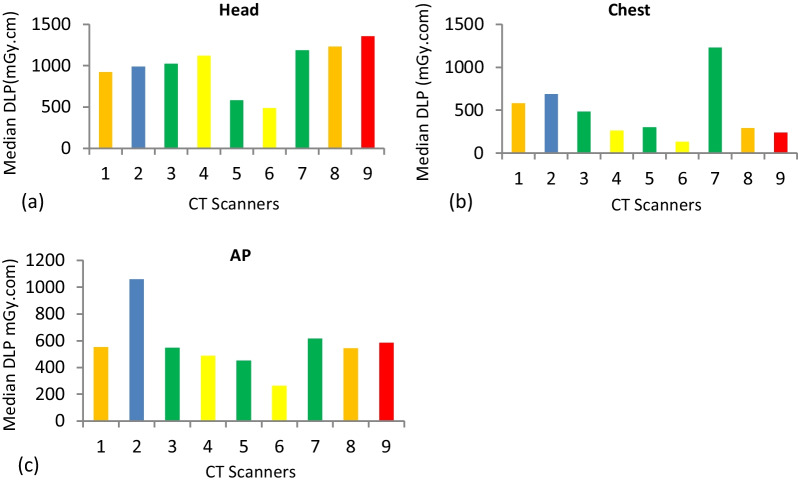
Median DLP distribution for examinations surveyed. **a** Median DLP for head. **b** Median DLP for chest **c** median DLP for AP

For each CT scanner and examination, descriptive statistics were calculated (Table 2). Based on ICRP 135 recommendation [6], mean, and median values of CTDI_vol_, and DLP were calculated for each facility (Figs. 1, 2, 3).

**Table 2:** Demographic distribution and characteristics of study population

CT scanner	Examination type	Age in Years				Sex			Weight in Kg			
		Min	Max	Mean	SD	M	F	Total	Min	Max	Mean	SD
1	AP	19	70	45	13.64	12	18	30	55	77	62.23	5.19
	Chest	20	84	48.67	21.19	20	10	30	50	76	60.4	6.54
	Head	22	68	40.9	13.14	17	13	30				
2	AP	23	83	49.53	16.83	14	16	30	45	79	64.53	8.63
	Chest	19	83	53.63	19.89	13	17	30	48	80	64.67	8.77
	Head	19	80	47.43	15.91	12	18	30				
3	AP	22	74	42.53	15.19	16	14	30	42	78	56.07	8.78
	Chest	22	75	41.73	16.22	22	8	30	45	77	63.83	5.62
	Head	24	68	40.9	13.14	20	10	30				
4	AP	24	85	52.37	14.79	14	16	30	52	79	63.4	7.19
	Chest	27	80	54.3	15.94	16	14	30	40	78	56.07	9.7
	Head	19	81	46.47	19.95	22	10	32				
5	AP	19	85	45.67	16.76	13	17	30	40	79	62.33	8.94
	Chest	23	72	44.87	12.55	17	13	30	48	80	64.37	9.3
	Head	19	64	37.57	14.32	13	17	30				
6	AP	19	83	41.1	17.66	22	8	30	49	80	70.43	7.89
	Chest	28	84	54.17	15.53	10	20	30	47	80	65.13	9.06
	Head	19	80	49.57	19.53	15	15	30				
7	AP	19	80	40.23	12.51	19	16	35	42	78	60.57	10.77
	Chest	19	65	47.54	14.74	11	24	35	41	76	57.23	8.26
	Head	22	73	49.74	15.56	18	17	35				
8	AP	19	75	43.76	15.09	13	22	35	41	76	56.09	9.66
	Chest	25	75	45.71	15.28	15	16	31	44	80	56.35	10.26
	Head	19	76	42.09	15.12	22	13	35				
9	AP	18	88	53	17.57	14	16	30	40	80	62.40	12.57
	Chest	21	82	44.7	14.53	12	18	30	41	80	58.63	12.18
	Head	21	75	40.53	15.77	19	11	30				

*SD* standard deviation, *Min* minimum, *Max* maximum

The DRLs were calculated using CT dose parameters, specifically the CTDI_vol_ and DLP values displayed on the CT scanner console and based on ICRP guidelines [6]. The 25th percentile, medians, and 75th percentiles from each median value were calculated for each of the examinations (See Figs. 2, 3). And the rounded third 75th percentiles were taken as DRLs.

## Results

The study performed for the most commonly practiced CT examination; the head, chest, and AP and proposed DRLs. Data were collected from three public hospitals, three private hospitals, and three diagnostic centers. The entire data set was acquired from helical scan mode. Table 1 lists the CT scanners used in our study. From nine CT scan facilities in Addis Ababa, the information was gathered from 431 (51.43%) male and 407(48.57%) female adult patients (See Table 2). The data gathered includes details about 282 (33.65%) head CT exams, 276 (32.94%) chest CT examinations, and 280 (33.41%) AP CT examinations (see Table 2).

The CT exposure parameters were examined using the data. The minimum tube voltages for the head, chest, and AP examinations were 80 kV, 80 kV, and 70 kV, respectively. The maximum tube voltage used across all examinations was 130 kV. Correspondingly, the mean tube current–time product (mAs) used for the head, chest, and AP were 276mAs (99.90), 158.76mAs (80.99), and 179.83mAs (62.04), respectively. The mean tube voltages used for the head, chest, and AP were 119.89 kV (5.16), 115.70 kV (13.21), and 115.39 kV (14.03), respectively. The pitches used for the head, chest, and AP examinations were 0.5–1, 0.55–1.5, and 0.5–1.5, respectively.

To compare dose values among the CT centers, descriptive statistics were computed for each center (Fig. 1a–f). The highest variation was seen in chest CT, with 329.50% and 619.85% variations between the minimum and maximum doses in CTDI_vol_ and DLP, respectively. While, smallest variances between the minimum and maximum dose values were seen in head CT, with 148.38% and 197.97% discrepancies for CTDI_vol_ and DLP, respectively (Fig. 1a–f).

The CTDI_vol_ and DLP between public hospitals, private hospitals, and diagnostic centers did not indicate any statistically significant mean differences (*p* > 0.05) per an independent-samples *t*-test.

This study identified identical CT scanner models, allowing protocol comparisons (Table 1). CT dose indicators were collected from three Philips 64-slice scanners, two Siemens 16-slice and one 128-slice scanners, two GE OPTIMA CT660 scanners, and one Neusoft 64-slice scanner. For Philips CT scanners with 64 slices, the discrepancy between the maximum and minimum mean values were 116.23%, 23.30%, and 63.44% for CTDI_vol_, and 152.45%, 211.08%, and 121.35% for DLP for head, chest, and AP, respectively.

DLP differences of 139.47%, 103.74%, and 83.52% were observed for head, chest, and AP between 16-slice Siemens scanners while 23.63%, 67.88%, and 9.70% DLP differences were observed for the head, chest, and AP, respectively, between GE 64-slice scanners (Fig. 1a–f).

For consistency, median values were used to compute DRLs as recommended by ICRP [6]. The CTDI_vol_ values of each CT scanner were calculated for each CT examination (Fig. 2a–c).

The DLP median values of each CT scanner were calculated for each CT examination (Fig. 3a–c).

From the median values of each CT facilities, the 25th percentile, median (50th percentile), and 75th percentile were calculated (Table 4). The median CTDI_vol_ values were 48.10, 7.58, and 10.21 for the head, chest, and AP CT examinations, respectively. As well as DLPs, the median values were 1022.15, 300.50, and 545.90 for CT examinations of the head, chest, and AP, respectively. The 75th percentile was determined using median values calculated from selected institutions and set as local DRLs.. The DRLs for CTDI_vol_ and DLP values established for this study; DRLs for CTDI_vol_: 53 mGy, 13 mGy and 16 mGy for head, chest, and AP respectively and the proposed DRLs for DLP are 1210 mGy.cm, 635 mGy.cm, and 822 mGy.cm for head, chest, and AP, respectively. The proposed DRLs were compared to the published local, regional, and international DRLs values.

### Chapter 4: Discussion

The public, private, and private diagnostic medical diagnostic centers that make up Ethiopia's healthcare system all offer medical diagnostic services. In his study data were prospectively collected from 282 head, 276 chest, and 280 AP CT examinations. The study's main objective of proposing local DRLs for commonly conducted investigations in Addis Ababa was met. And the DRLs set for CTDI_vol_ are: 53 mGy, 13 mGy, and 16 mGy for head, chest, and AP, respectively. The DRLs for DLP are: 1210 mGy.cm, 63 mGy.cm, and 822 mGy.cm for head, chest, and AP, respectively.

There exist dose variations for the same examination among CT facilities within the city. These variations may result from user selections of parameters such as kVp, mAs, and pitch as well as manufacturer-specific variations in the design of CT equipment. Comparing the mean values of the facilities; the head CT examination showed the smallest differences between the minimum and maximum dose values (23.42, 58.17) for CTDI_vol_ (see Fig. 1a). Chest CT scans showed the largest variation, with mean maximum and minimum values for CTDI_vol_ and DLP of 3.22 and 13.83, and 134.95 and 971.44 respectively. This greatest variation in chest CTDI_vol_ occurred between CT scanner 7 and 9; may be caused by the CT protocols used by the two facilities. For instance CT scanner 7 applied 250 mAs on average for the chest examination while CT scanner 6 applied 70 mAs (see Table 3). Similar CT scanners also showed dose variations. The biggest dose variation was seen in the head DLP of two Philips 64-slice CT scanners, facilities 5 and 7. The discrepancy may have been caused by the different mAs used by the two facilities (Facility 5 used 165 mAs whereas facility 7 used 250mAs).

**Table 3:** CT exposure parameters by anatomic region for adult examination

Examination	Protocol	CT facilities								
		1	2	3	4	5	6	7	8	9
Head	kVp	120	120	120	130	100	110	120	120	120
	mAs	320	400	350	250	165	150	400	350	160
	Pitch	1.37	0.67	1.0	0.55	1.0	1.0	0.6	0.6	0.5
Chest	kVp	120	120	120	130	120	110	120	120	80
	mAs	250	250	300	100	165	125	250	300	70
	Pitch	0.98	0.86	1.0	1.5	0.75	1.0	0.8	0.6	0.6
AP	kVp	120	120	120	130	120	110	120	120	80
	mAs	300	250	250	125	160	150	200	250	90
	Pitch	0.5	0.86	1.0	1.5	0.75	1.0	1.1	0.6	0.55

This study's established DRLs were compared to latest (2016–2022) local, regional, and international DRLs (Table 5). We have compared the current study’s proposed DRLs of the current study with other DRLs established in Egypt [17], Morocco [18], USA [19], UK [13], Australia [14], and other selected studies (Table 4). A study done in Jimma University Medical Center (Southwest Ethiopia) established a local DRLs values for CTDI_vol_ as 42.97 mGy, 7.76 mGy, and 10.86 mGy for head, chest, and A, respectively, and for DLP as 1364.15 mGy.cm, 368.96 mGy.cm, and 1568.96 mGy.cm for head, chest, and AP, respectively20. In comparison to this study, the current study shows greater CTDI_vol_ levels across all examinations and lower DLP values overall, with the exception of the chest examinations. The variation might be accounted to the number of CT scanners examined.

**Table 4:** Established LDRLs for the Three Examinations

	CTDI_vol_ (mGy)			DLP (mGy.cm)		
	Head	Chest	AP	Head	Chest	AP
Mean	43.27	9.14	11.10	988.52	432.76	635.09
Median	48.10	7.58	10.21	1022.15	300.50	545.90
SD	12.70	5.42	5.54	290.02	255.10	306.80
Range	34.00	17.06	15.00	868.22	786.12	966.84
Minimum	23.65	2.97	5.04	487.28	131.68	263.36
Maximum	57.65	20.03	20.04	1355.50	917.80	1230.20
Percentiles						
25	29.34	4.47	6.16	752.13	249.54	469.51
50	48.10	7.58	10.21	1022.15	300.50	545.90
75	53	13	16	1210	635	822

Comparing current DRLs with those of other African countries, the largest discrepancy for DRLs of DLP for AP was found between this study (822 mGy.cm) and those of Kenya (1845 mGy.cm) (Table 5). However, the proposed DRLs values are comparable to the AP DRLs of CTDI_vol_ proposed for the regional values set for the four African countries (16 mGy vs.15.7 mGy). Although the DRL values indicated by this study do not differ considerably from international DRL values (see Table 5), the large dose distribution between CT facilities in Addis Ababa, Ethiopia implies that CT studies should be optimized.

**Table 5:** Comparison of the established DRLs

Country	Head		Chest		AP	
	CTDI_vol_ (mGy)	DLP (mGy.cm)	CTDI_vol_ (mGy)	DLP (mGy.cm)	CTDI_vol_ (mGy)	DLP (mGy.cm)
Ethiopia (current study) (2022)	53	1210	13	635	16	822
Ethiopia(Jimma University Medical Center) (2020) [20]	42.97	1364.15	7.76	368.96	10.86	1568.96
Morocco (2021) [18]	57.4	1020	12.3	632	10.9	714
Nigeria (2018) [21]	61	1310	17	735	20	1486
Kenya (2016) [22]	61	1612	19	895	20	1845
Egypt (2017) [17]	30	1360	22	420	31	1325
Uganda (2022) [23]	56	1260	7.8	377	12.5	1418
Japan (2020) [24]	77	1350	13	510	18	880
USA (2017) [19]	57	1011	15	545	20	1004
Canada (2016) [16]	79	1302	14	521	1818	874
Australia (2020) [14]	52	880	10	390	13	600
UK (2019) [13]	46	804	9	328	12	621
Regional DRLs (Ghana, Kenya, Namibia, and Senegal) (2021) [25]	60.9	1259	15.2	544	15.7	737

There are various potential reasons why CT doses vary between institutions. It is believed that various CT techniques are the primary. Doses can be reduced by lowering kVp, decrease tube current and increasing pitch factor [26, 27]. Furthermore, staff training and awareness of CT scan technical characteristics have a considerable impact on patient dose [28]. The scanning protocols used by operators in different countries differ, and these differences are dependent on the training and experience of the operators. Abdulkadir et al. [29] discovered that radiographers had insufficient knowledge of DRLs, with a specific knowledge gap in the implementation of DRL local dose assessment and optimization.

This study's unique contribution is the establishment of the first suggested local DRLs using data from multiple facilities. The findings indicate that there is a significant opportunity for improvement in CT dose optimization among the facilities in the city using the proposed DRLs. Although previously proposed institution-based LDRLs [20], NDRLs have yet to be developed for the country, and these proposed LDRLs will play a unique role in NDRL development.

### Limitations

This study had several limitations. First, this work was relied on the accuracy of the CT scanners' displayed CTDI_vol_ and DLP; the authors did not utilize any other methods to confirm the accuracy. Second, since, the scan length were not recorded as required in all facilities, its impact on DLP values were not discussed.

### Conclusion and recommendation

Local DRLs for three of the most common CT examinations in Addis Ababa were proposed based on a city-wide assessment of nine facilities. The authors recommend that CT facilities use the proposed DRLs as a reference to conduct a continuous audit of their CT dose and the appropriateness of their scanning parameters in order to avoid giving patients excessively high doses. The study's findings should be communicated to CT facilities and users so that they are informed of their activities and practices followed in other centers. The NDRLs must be established as soon as possible, and dose optimization techniques must be adopted across the country. Furthermore, the authors suggest that the DRLs should be included in curricula and that intensive training on specific equipment be required because the operator's experience is crucial to the optimization of CT dose. The authors also called for ongoing radiographer training that placed more of an emphasis on dose optimization and institutionally based dose evaluation.

## Supplementary Information

**Additional file 1. Appendix A.** Survey Questionnaire.

## Data Availability

All the necessary data and materials have been included in this manuscript.

## Abbreviations

ALARA: As low as reasonably achievable
AP: Abdominopelvic
CTDI_vol_: Volumetric Computed Tomography Dose Index
CT: Computed tomography
DLP: Dose length product
DLR: Diagnostic reference levels
ICRP: International Commission of Radiological Protection
LDRL: Local diagnostic reference level
NDRL: National diagnostic reference level

## References

[1] Schauer DA, Linton OW (2009). National Council on Radiation Protection and Measurements report shows substantial medical exposure increase. Radiology.

[2] FDA. Computed Tomography (CT). [cited 2022 Aug 8]. Available from: https://www.fda.gov/radiation-emitting-products/medical-x-ray-imaging/computed-tomography-ct

[3] Shao YH, Tsai K, Kim S, Wu YJ, Demissie K (2020). Exposure to tomographic scans and cancer risks. JNCI Cancer Spectr.

[4] UNEP. Radiation effects and sources: What is radiation? What does radiation do to us? Where does radiation come from? 2016. 1–68 p.

[5] Hall EJ, Brenner DJ (2008). Cancer risks from diagnostic radiology. Br J Radiol.

[6] ICRP, 2017. Diagnostic reference levels in medical imaging. ICRP Publication 135. Ann. ICRP 46(1) 10.1177/0146645317717209 29065694

[7] Tsapaki V. Radiation dose optimization in diagnostic and interventional radiology: Current issues and future perspectives. Phys Medica. 2020;79(September):16–21. Available from: 10.1016/j.ejmp.2020.09.015 10.1016/j.ejmp.2020.09.015 33035737

[8] ICRP. Radiological protection and safety in medicine. A report of International Commission on Radiological Protection. Ann ICRP. 1996;26:1–47. 8911634

[9] Cho PK. Diagnostic Reference Levels. Image Wisely. 2010.

[10] IAEA. Radiation protection and safety in medical uses of ionizing radiation: specific safety guide. 2018;340.

[11] European Comission. European guidelines on quality criteria for computed tomography European guidelines on quality criteria. Eur 16262 En. 1999;1–71.

[12] Muhammad NA, Abdul Karim MK, Abu Hassan H, Ahmad Kamarudin M, Ding Wong JH, Ng KH (2020). Diagnostic reference level of radiation dose and image quality among paediatric CT examinations in a Tertiary Hospital in Malaysia. Diagnostics (Basel).

[13] UKHSA-RCE-1: Doses from computed tomography (CT) exams in the UK 2019 review. 2019.

[14] Lee KL, Beveridge T, Sanagou M, Thomas P (2020). Updated Australian diagnostic reference levels for adult CT. J Med Radiat Sci.

[15] Japan Medical Imaging and Radiological Systems Industries Association, National Institutes for Quantum and Radiological Science and Technology. National Diagnostic Reference Levels in Japan (2020). 2020;1–22.

[16] Canadian Computed Tomography Survey – National Diagnostic Reference Levels, by Graeme M Wardlaw, PhD and Narine Martel, MSc. Ottawa: Health Canada, May 2016. Pub. No. 160038.

[17] Salama DH, Vassileva J, Mahdaly G, Shawki M, Salama A, Gilley D, et al. Establishing national diagnostic reference levels (DRLs) for computed tomography in Egypt. Physica Medica, 39. Phys medica. 2017;39:16–24. 10.1016/j.ejmp.2017.05.050 28711184

[18] El Mansouri M, Talbi M, Choukri A, Nhila O, Aabid M (2022). Establishing local diagnostic reference levels for adult computed tomography in Morocco. Radioprotection.

[19] Kanal KM, Butler PF, Sengupta D, Bhargavan-Chatfield M, Coombs LP, Morin RLUS (2017). Diagnostic reference levels and achievable doses for 10 adult CT examinations. Radiology.

[20] Zewdu M, Kadir E, Tesfaye M, Berhane M (2021). establishing local diagnostic reference levels for routine computed tomography examinations in Jimma University Medical Center South West Ethiopia. Radiat Prot Dosimetry.

[21] Ekpo EU, Adejoh T, Akwo JD, Emeka OC, Modu AA, Abba M, Adesina KA, Omiyi DO, Chiegwu UH (2018). Diagnostic reference levels for common computed tomography (CT) examinations: results from the first Nigerian nationwide dose survey. J Radiol Prot.

[22] Korir GK, Wambani JS, Korir IK, Tries MA, Boen PK (2016). National diagnostic reference level initiative for computed tomography examinations in Kenya. Radiat Prot Dosimetry.

[23] Erem G, Ameda F, Otike C, Olwit W, Mubuuke AG, Schandorf C, et al. Adult computed tomography examinations in Uganda: towards determining the National Diagnostic Reference Levels. BMC Med Imaging. 2022;1–12. Available from: 10.1186/s12880-022-00838-x 10.1186/s12880-022-00838-x PMC9188687 35690743

[24] Kanda R, Akahane M, Koba Y, Chang W, Akahane K, Okuda Y, Hosono M (2021). Developing diagnostic reference levels in Japan. Jpn J Radiol.

[25] Uushona V, Boadu M, Nyabanda R, Diagne M, Inkoom S, Issahaku S (2022). Establishment of regional diagnostic reference levels in adult computed tomography for four African Countries: a preliminary survey. Radiat Prot Dosimetry.

[26] Callahan MJ (2011). CT dose reduction in practice. Pediatr Radiol.

[27] Maldjian PD, Goldman AR (2013). Reducing radiation dose in body CT: a primer on dose metrics and key CT technical parameters. Am J Roentgenol.

[28] Lange I, Alikhani B, Wacker F, Raatschen HJ. Intraindividual variation of dose parameters in oncologic CT imaging. PLoS One. 2021;16(4 April):1–10. Available from: http://dx.doi.org/10.1371/journal.pone.0250490 10.1371/journal.pone.0250490 PMC8064522 33891632

[29] Abdulkadir MK, Piersson AD, Musa GM, Audu SA, Abubakar A, Muftaudeen B, et al. Assessment of diagnostic reference levels awareness and knowledge amongst CT radiographers. Egypt J Radiol Nucl Med. 2021;52(1).

