# Supplementary material for: Establishment of local diagnostic reference levels for common adult CT examinations: a multicenter survey in Addis Ababa
Source: BMC Med Imaging. 2023 Jan 9;23:6. doi: 10.1186/s12880-023-00963-1 (PMC9830915; doi:10.1186/s12880-023-00963-1)
Supplement: Supplementary file 1 — Additional file 1. Appendix A. Survey Questionnaire. [file 12880_2023_963_MOESM1_ESM.docx]

**Full-title:** Establishment of local diagnostic reference levels for common adult CT examinations: A multicenter survey in Addis Ababa

Marema Jebessa Kumsa^1^*, Teklehaimanot Mezgebe Nguse^1^, Haleluya Biredaw Ambessa^1^, Tesfaye Tefera Gele^1^, Wondemu Geteye Fantaye^1^, Seife Teferi Dellie^2^

# Appendices

## Appendix A

### Survey Questionnaire

| **Diagnostic Reference Level for Adult Computed Tomography Examinations** | | | | | | | | | | | | | | | | | | | |
| --- | --- | --- | --- | --- | --- | --- | --- | --- | --- | --- | --- | --- | --- | --- | --- | --- | --- | --- | --- |
|  | **Name of Facility:______________________** | | | | |  |  |  | **Manufacturer:_________________________** | | | | | |  | **kVp: _______________** | | | |
|  | **Year of Manufacture___________________** | | | |  |  |  |  | **Year of Installation: _____________________** | | | | | |  |  | | | |
|  | **Capacity (slice number):_____________** | | | |  |  |  |  | **Tube Current Modulation: ___________Y/N** | | | | | |  |  |  |  |  |
|  |  |  | Patient Details | | | |  |  | Exposure Parameters | | | | | | | | Dose Data Displayed | | |
| Patient No. | Type of Exam | Date of Exam | Sex | Age | weight (kg) | Height (cm) | Sequence number | Contrast material | kV | mA | mAs | Total scan time (s) | Pitch | Beam width | Scaning range (cm) | Scanning Mode (A/H) | CTDIvol (mGy) | Reference phantom (16 cm or 32 cm) | Total DLP (mGy.cm) |
| **1** |  |  |  |  |  |  |  | Scout |  |  |  |  |  |  |  |  |  |  |  |
|  |  |  |  |  |  |  |  | WOC |  |  |  |  |  |  |  |  |  |  |  |
|  |  |  |  |  |  |  |  | WC |  |  |  |  |  |  |  |  |  |  |  |
| **2** |  |  |  |  |  |  |  | Scout |  |  |  |  |  |  |  |  |  |  |  |
|  |  |  |  |  |  |  |  | WOC |  |  |  |  |  |  |  |  |  |  |  |
|  |  |  |  |  |  |  |  | WC |  |  |  |  |  |  |  |  |  |  |  |
| **3** |  |  |  |  |  |  |  | Scout |  |  |  |  |  |  |  |  |  |  |  |
|  |  |  |  |  |  |  |  | WOC |  |  |  |  |  |  |  |  |  |  |  |
|  |  |  |  |  |  |  |  | WC |  |  |  |  |  |  |  |  |  |  |  |
|  |  |  |  |  |  |  |  | WC |  |  |  |  |  |  |  |  |  |  |  |

**Abbreviations**

**DRL = Diagnostic Reference Level**

**FOV = Field of view**

**CTDI = Computed tomography dose index**

**DLP = Dose length product**

**WC=with contrast**

**WOC= without contrast**

**A = Axial**

**H = Helical**
